# Evaluation of Serum 25-Hydroxyvitamin D as a Predictor of Carotid Intima-Media Thickness and Carotid Total Plaque Area in Nonsmokers: The Tromsø Study

**DOI:** 10.1155/2013/305141

**Published:** 2013-11-20

**Authors:** Elena Kamycheva, Stein Harald Johnsen, Tom Wilsgaard, Rolf Jorde, Ellisiv B. Mathiesen

**Affiliations:** ^1^Geriatric Section, Division of Internal Medicine, University Hospital of North Norway 9038 Tromsø, Norway; ^2^Tromsø Endocrine Research Group, Institute of Clinical Medicine, University of Tromsø, 9037 Tromsø, Norway; ^3^Department of Community Medicine, University of Tromsø, Breivika, 9037 Tromsø, Norway; ^4^Brain and Circulation Research Group, Department of Clinical Medicine, University of Tromsø, Breivika, 9037 Tromsø, Norway; ^5^Department of Neurology and Neurophysiology, University Hospital of North Norway, 9038 Tromsø, Norway

## Abstract

*Objective*. Altered calcium homeostasis has been linked to increased intima-media thickness (IMT) and plaques. We aimed to investigate whether serum 25-hydroxyvitamin D (25(OH)D) and serum calcium are associated with IMT and plaques in nonsmoking population. *Methods*. Ultrasound of the right carotid artery with the measurements of IMT and plaques was performed in 4194 nonsmoking subjects with available measurements of serum 25(OH)D and total calcium. Linear regression was applied to study the linear relationships between variables. Multinomial logistic regression was used to evaluate predictors of increased IMT and total plaque area (TPA), adjusted for age, body mass index, systolic blood pressure, and total cholesterol. *Results*. There was no significant linear relationship between mean IMT, TPA, and either serum 25(OH)D or total serum calcium. One SD increase in serum 25(OH)D was independently associated with increased odds of being in the highest quartile of IMT in men (OR 1.30, 95% CI 1.12, 1.51). In women, 1 SD increase in serum 25(OH)D was independently associated with increased risk of being in the upper tertile of TPA (OR 1.15, 95% CI 1.01, 1.33). *Conclusions*. Impaired calcium homeostasis has no consistent association with mean IMT and TPA; however, increased serum 25(OH)D may predict subclinical atherosclerosis in nonsmokers.

## 1. Introduction

Carotid atherosclerosis is a progressive, multifactorial artery disease, associated with high risks of morbidity and mortality [1]. Subclinical carotid atherosclerosis is an acknowledged challenge in primary stroke prevention [2]. Several underlying environmental and modifiable factors, such as increased, elevated systolic blood pressure (SBP), dyslipidemia, smoking, and increased body mass index (BMI), play a significant role in the progression of the atherogenic process [2]. Carotid intima-media thickness (IMT) and plaque assessment by cervical ultrasonography is a noninvasive, feasible, and accurate method for detecting asymptomatic carotid atherosclerosis [3]. It is also largely used for risk assessment in primary prevention and in clinical trials as a surrogate endpoint for cardiovascular and cerebral events [3]. Both IMT and total plaque area (TPA) are associated with an increased risk of ischemic stroke and coronary heart disease [3, 4]. It is well established that the presence of carotid plaques represents advanced carotid atherosclerosis, often symptomatic, whereas IMT can be assessed in the absence of focal plaques and symptoms [4, 5].

Altered calcium homeostasis, including vitamin D deficiency, was recently associated with subclinical atherosclerosis [6] and low bone mineral density, which may predict risk of stroke in women [7]. Though the main role of vitamin D is the maintenance of mineral homeostasis and bone health [8], recent large epidemiological studies have linked vitamin D insufficiency to cardiovascular diseases, infections, and even cancer [9–11]. Furthermore, low serum levels of 25-hydroxyvitamin D (25(OH)D), the metabolite which is used to assess a subject's vitamin D status [12], are associated with insulin resistance and metabolic syndrome [13, 14].

In the fourth survey of population-based Tromsø Study in North Norway in 1994-1995, ultrasonography of the right carotid artery and measurements of serum 25(OH)D and total calcium were performed in a large subgroup, thus allowing us to evaluate the potential relationship between vitamin D status and both IMT and plaques.

## 2. Materials and Methods

### 2.1. Study Population

The Tromsø Study is a population-based health survey, which was performed for the first time in 1974 [15]. In the fourth survey of the Tromsø Study in 1994-1995, all men and women at age >24 years and living in the municipality of Tromsø, North Norway, were invited to participate. All subjects aged 55–74 years and random 5–10% samples of the other age groups were invited to a second visit for a more extensive examination which included carotid ultrasound examination. Height and weight were measured while the subjects wore light clothing and no shoes. Body mass index (BMI) was defined as weight (kg) divided by squared height (m^2^). Blood pressure and heart rate were measured with an automatic device (Dinamap Vital Signs Monitor 1846, Critikon Inc., Tampa, FL) and the mean measurement of the second and the third measurements was used in the statistical analysis. Blood samples were drawn in a nonfasting state. Serum levels of 25(OH)D were analysed by immunometry (electrochemiluminescence immunoassay), using an automated clinical chemistry analyser (Modular E170; Roche Diagnostics). According to the manufacturer, the assay has a total coefficient of variation (CV) ≤7.8% as judged at any of the three different concentrations (48.6, 73.8 and 177.0 nmol/L). Analytical sensitivity was 10 nmol/L and a population-based reference range was 27.7–107.0 nmol/L for adults as provided by the manufacturer. This analysis has been approved by the Norwegian Accreditation Authority. With this assay, it has recently been described that smokers had 15–20% higher serum 25(OH)D than that of nonsmokers [16]. However, the same pattern was not observed when using other immunological and liquid-chromatography mass spectrometry methods [16]. This discrepancy is still lacking an explanation, and to avoid this bias, we have included only nonsmokers in our study cohort. Serum total cholesterol was analyzed by enzymatic colorimetric methods (CHOD-PAP; Boehringer Mannheim). The determination of serum calcium was performed on Hitachi 917 with reagents from Boehringer Mannheim, reference range 2.20–2.60 mmol/L and CV is 2%. Lipid levels were measured twice with an interval of 4 to 12 weeks, and the averages of these values were used in the analysis.

### 2.2. Questionnaires

Information about smoking habits, angina pectoris, previous myocardial infarction, and stroke was collected from self-administered questionnaires. Smokers were classified as current smokers or nonsmokers. 

### 2.3. Measurements of IMT and Carotid Plaques

Carotid ultrasound was performed as previously described in detail [4]. In short, the examination of the right carotid artery was performed in the supine position with a high-resolution Acuson 128XP/10 ART upgraded scanner (Mountain View, CA) equipped with a linear transducer with 7 MHz in B mode and 5 MHz in pulsed-Doppler mode. IMT was measured in 10 mm segments in three locations of the carotid artery: the near and far wall of the common carotid artery (CCA) and the far wall (FW) of the bifurcation. The loss of parallel configuration of the near and far walls of the CCA served as a reference point for the start of the carotid bifurcation. Three frozen images from each segment were stored in high-resolution videotapes. The ultrasonic images were analyzed offline with a computerized technique for automatic ultrasonic image analysis. The average IMT from three preselected images was calculated for each location. Plaques were included in the measurements of IMT if they were located in areas predefined for IMT registration. The reproducibility of the ultrasound measurements was acceptable [17].

A plaque was defined as a localized protrusion into the vessel lumen with thickening of the vessel wall of >50% compared to the adjacent IMT. Frozen images were recorded for each plaque and digitalized for the further examination. Six locations of the carotid artery were examined for plaque presence, the far and near walls of the CCA, the bifurcation (bulb), and the ICA. The area of each plaque was outlined manually with automatic calculation of plaque area. TPA was calculated as the sum of plaque areas in all six locations.

### 2.4. Statistics

Continuous variables are presented as mean ± standard deviation (SD), unless otherwise stated. All continuous variables were standardized prior to inclusion in the linear and logistic regression analyses. The distribution of all variables was determined by measuring skewness and kurtosis and assessments of normal Q-Q plots of standardized variables. TPA was square root transformed to obtain normal distribution. Analyses of the scatter plots and linear regression model were used to assess the relation between serum 25(OH)D and the measurements of carotid atherosclerosis (mean IMT, TPA, and plaques). Strata of mean IMT (quartiles) and TPA (no plaques or tertiles of TPA) were created. A multinomial logistic regression model was applied to assess the effect of serum 25(OH)D and total calcium across strata of mean IMT (using first quartile as a reference) and TPA (using no plaques as a reference group). Continuous variables: (age, BMI, SBP, total cholesterol) and categorical variable season (summer, fall, winter and spring) of vitamin D sampling were included in the respective regression models as covariates. Since men and women have significantly different mean IMT, genders were analysed separately. Comparisons between groups were analysed with ANOVA. The analysis of the whole population was performed without the exclusion of smokers. We have identified 5 groups of 25(OH)D concentration (<30.0 nmol/L, 30.0–49.9 nmol/L, 50.0–74.9 nmol/L, 75.0–100.0 nmol/L, and >100.0 nmol/L). Using the cutoff of the highest mean IMT quartile as a clinical endpoint and the 25(OH)D groups as predictors, the logistic regression was applied, adjusted for age, BMI, SBP, season for vitamin D sampling, total cholesterol, and smoking status. All tests were done two sided, and a *P* value < 0.05 was considered statistically significant. The data was analysed with IBM SPSS Statistics Version 19 (SPSS Inc., Chicago, IL, USA).

### 2.5. Ethics

The study was approved by the Regional Committee for Medical and Health Research Ethics, the Data Inspectorate, and the Norwegian Directorate of Health.

### 2.6. Results

Ultrasound examination of the right carotid artery was performed in 6727 subjects (77% of the eligible) and in 6230 of those serum 25(OH)D measurements were available. After smokers were excluded, 1994 men and 2200 women were included in the final analysis.

The characteristics of the study population are shown in Table 1.

There were no linear associations between serum 25(OH)D and either mean IMT or TPA, when adjusted for age, BMI, systolic blood pressure, total cholesterol, and season of blood sampling (Tables 2 and 3). To study the threshold effects, we compared the ORs, across quartiles of IMT and tertiles of TPA, with the lowest quartile of IMT and the no plaque group as reference groups, respectively. In males, a 1 SD increase in serum 25(OH)D was associated with a 30% increased odds for having IMT >1.00 mm, compared to the reference group (Table 4). No effect of serum 25(OH)D was found on TPA. In females, no independent association was found between serum 25(OH)D and mean IMT. However, a 1 SD increase in serum 25(OH)D was associated with a 15% higher odds of having TPA >18.07 mm^2^ versus the absence of plaques (Table 4). 

In crude analyses, the increase in total calcium was associated with the increased odds being in higher levels of mean IMT and TPA (Table 5). When adjusted for age, BMI, SBP, and total cholesterol, neither of these associations was significant (Table 5).

The logistic regression analysis with 25(OH)D groups as the predictor variable and the sex-specified highest quartile of mean IMT as an outcome variable was performed in the whole population, without the exclusion of smokers as well as in nonsmokers. Neither of the 25(OH)D groups turned out to be a significant predictor of the highest quartile of mean IMT in both genders in the whole population, whereas male nonsmokers with serum 25(OH)D >100 nmol/L were at 77% higher risk to have mean IMT >1.00 mm, compared to those with serum 25(OH)D within the range 75–100 nmol/L, but not statistically significant, Table 6. 

## 3. Discussion

In our large subgroup of non-smoking general population, men had a significantly higher mean IMT compared to women. Impaired calcium homeostasis was not found to have a significant association with mean IMT and TPA. An increase in serum 25(OH)D was independently associated with increased odds of being in the highest quartile of mean IMT >1.00 mm in males and TPA >18.07 mm^2^ in females. No associations were observed between total calcium and mean IMT or TPA. When smokers were included in our cohort, serum 25(OH)D was no longer a significant predictor of higher mean IMT in either gender.

Male gender, increased BMI, and smoking status are well-established risk factors of carotid stenosis (1, 2), and our finding of higher IMT in men supports the results in other studies [18]. Recently, a significant contributing impact of deranged calcium homeostasis with decreased serum 25(OH)D and high calcium-phosphorus product has been reported in the observational studies [6, 11]. There is also some evidence that the active vitamin D metabolite, 1,25 dihydroxyvitamin D (1,25(OH)_2_D), might exert beneficial physiological effects in both vascular smooth muscle cells and the vascular endothelium [19]. In an observational study by Al Mheid et al. [20] on 554 healthy volunteers, lower levels of serum 25(OH)D were associated with vascular stiffness and endothelial function. However, to our knowledge, none of the randomised trials have supported any causal association between vitamin D and arterial disease. In a study by Stricker et al. [21], 62 patients with peripheral artery disease without vitamin D deficiency received either a single dose of 100,000 IU cholecalciferol or placebo in an observation period of 1 month. No influence of vitamin D supplementation was demonstrated on either cardiovascular surrogate parameters (blood pressure, aortic stiffness, and endothelial function) or parameters of thrombosis (markers of thrombin generation and markers for fibrinolysis and platelet activation). We have previously reported an association between the thrombogram parameters and serum levels of 25(OH)D [22]. However, similarly to the study by Stricker et al. [21], no influence of vitamin D supplementation on those parameters was seen. 

Inconsistent results on vitamin D and peripheral atherosclerosis burden were also demonstrated in the disease state. Thus, in 390 diabetic patients, the hypovitaminosis D (defined as serum 25(OH)D <15 nmol/L) was associated with greater IMT [23]. Whereas, in a study by Yiu et al. [24], daily oral supplementation of 5000 IU vitamin D3 was given to 100 patients with type II diabetes during a 12-week period, and no improvement in vascular function was seen at the end of the study. 

Our study is, to our knowledge, the largest observational study on the associations between serum 25(OH)D and markers of carotid atherosclerosis. No convincing associations were observed between serum 25(OH)D and intima-media thickness or plaques in a large subgroup of nonsmokers. The findings are consistent with other recent reports [25–27].

However, there is accumulating evidence that insufficient vitamin D status has adverse effects on the metabolic profile [13] and may be associated with the severity of arterial disease [28]. The potential role of vitamin D in atherogenesis, if any, is not completely known. It has also been speculated that vitamin D may possess some antithrombotic propensities [29], although no effects of vitamin D supplements were found on the markers of thrombosis [21, 22]. In animal models, oral supplementation with vitamin D reduced the formation of atherosclerotic plaques by the suppression of T lymphocytes [30]. Moreover, in a study by Pilz et al. [31], low serum 25(OH)D and 1,25(OH)_2_D were independent predictors of fatal stroke in more than 3000 subjects who were routinely referred to coronary angiography. It is hard to find an explanation for such discrepant findings. One of the possible explanations may be the existence of strong confounders that could either distort the association between vitamin D and atherosclerosis towards or away from the null hypothesis [32]. Smoking status and total calcium might be considered as some of these confounders. Alternatively, in studies where the regression analysis is adjusted for smoking status, smoking could still be the nuisance variable that distorts the association between exposure (serum 25(OH)D levels) and outcome (increased IMT and/or plaques) and either magnifies or decreases the statistical associations. In our study, we excluded all smokers and created separate statistical models for vitamin D and total calcium, thus combating powerful confounders.

Our findings of possible harmful effects of vitamin D on IMT and plaques in general non-smoking population are to our knowledge novel. Even though the findings should be interpreted with caution, a significant 30% higher risk of having mean IMT in the fourth quartile versus first quartile per 1 SD increase in 25(OH)D in males is hard to ignore. Moreover, the serum 25(OH)D concentrations >100.0 nmol/L might be potentially harmful towards the mean thickness of intima media in non-smoking males. Noteworthy, Zittermann et al. [33] reported a significant adverse effect of an increased serum 25(OH)D (>100 nmol/L) in a selected group of patients, undergoing heart surgery. The authors have demonstrated the U-shaped unadjusted association between preoperatively measured 25(OH)D and composite adverse outcome in patients scheduled for cardiac surgery. This recent finding and our results of a possible harmful impact of increased 25(OH)D definitely require further investigations and confirmative studies.

Our study has several limitations. First of all, smoking status was self-reported; thus biased self-reporting could have affected the inclusion cohort. Secondly, we measured IMT and plaques only in the right carotid artery, whereas measurements in both carotid arteries might have been more representative. And finally, we did not have the opportunity to adjust for serum parathyroid hormone (PTH), vitamin D binding protein (DBP), and circulating 1,25(OH)_2_D since the measurements of serum PTH were not available for the majority of our study participants and we did not measure DBP in our study. Another major shortcoming is that we were unable to measure the circulating 1,25(OH)_2_D levels, as in previous studies an inverse association between 1,25(OH)_2_D and coronary calcification was demonstrated [34]. On the other hand, we did include a substantial amount of subjects, and we included the analysis of total calcium and eliminated the major confounder smoking. We also adjusted the measurements for age, BMI, and total cholesterol, and the results of our large cross-sectional study are confirmative to previous reports [25–27]. 

## 4. Conclusions

In conclusion, we demonstrated no consistent association between serum 25(OH)D and mean IMT and TPA in a large group of non-smoking men and women. However, increment in serum 25(OH)D was associated with the elevated risk of being in the highest quartile of mean IMT in males and of having the largest TPA in women in our fairly vitamin D sufficient population. The clinical impact of vitamin D status for carotid artery disease prevention is most likely scanty, and potential clinical trials on subjects without vitamin D deficiency should be performed with caution. 

## Figures and Tables

**Table 1:** Characteristics of the study population. The 4th Tromsø Study, 1994-1995.

Variables	Men, *n* = 1994	Women, *n* = 2200
Age, years	60.5 ± 9.7	61.8 ± 9.8
History of cardiovascular disease, *n* (%)		
Heart attack	205 (10.2)	70 (3.2)
Angina	237 (11.8)	192 (8.7)
Stroke	64 (3.2)	56 (2.5)
BMI, kg/m^2^	26.5 ± 3.2	26.7 ± 4.5
Serum 25(OH)D, nmol L^−1^	54.8 ± 16.6	50.0 ± 16.7*
Total cholesterol, mmol L^−1^	6.5 ± 1.2	6.9 ± 1.3*
Total calcium, mmol L^−1^	2.37 ± 0.10	2.38 ± 0.11
Mean IMT, mm	0.90 ± 0.20	0.84 ± 0.17**
TPA^†^, mm^2^	12.1 ± 19.4	7.6 ± 13.0

^†^Subjects without plaque are not excluded.

*P < 0.05.

**P < 0.01.

**Table 2:** Serum 25(OH)D (means ± SD) in quartiles of mean IMT in men and regression coefficients (*β*) with their respective 95% confidential intervals from linear regression models. The 4th Tromsø Study, 1994-1995.

Mean IMT quartiles, mm (*N*)	Serum 25(OH)D^¶^, nmol L^−1^	Total calcium, mmol L^−1^
Men, *n* = 1994		
<0.76 (500)	53.7 ± 16.1	2.38 ± 0.10
0.76–0.87 (498)	55.5 ± 16.8	2.37 ± 0.10
0.88–1.00 (495)	54.2 ± 16.4	2.37 ± 0.10
>1.00 (501)	56.0 ± 17.2	2.36 ± 0.11
*P* for trend	NS	NS
*β* unadjusted (95% CI)^*₤*^	0.04 (−0.01, 0.09)	−0.03 (−0.08, 0.02)
*β* adjusted^†^ (95% CI)^*₤*^	0.05 (0.00, 0.01)	−0.10 (−0.05, 0.03)

Women, *n* = 2200		
<0.72 (554)	51.9 ± 18.0	2.36 ± 0.11
0.72–0.81 (547)	50.1 ± 17.0	2.38 ± 0.12
0.82–0.93 (548)	50.0 ± 16.0	2.39 ± 0.11
>0.93 (551)	47.9 ± 16.7	2.40 ± 0.11
*P* for trend	<0.001	<0.001
*β* unadjusted (95% CI)^*₤*^	−0.08 (−0.14, −0.05)	0.12 (0.08, 0.17)*
*β* adjusted^†^ (95% CI)^*₤*^	0.02 (−0.02, 0.06)	0.21 (−0.02, 0.06)

^¶^Adjusted for season of blood testing.

*P < 0.01.

^*₤*^Mean IMT as the dependent variable in the linear regression model.

^†^Adjusted for age, BMI, systolic blood pressure, and total cholesterol.

**Table 3:** Serum 25(OH)D (means ± SD) in the TPA groups in men and women and regression coefficients (*β*) with their respective 95% confidential intervals from linear regression models. The 4th Tromsø Study, 1994-1995.

TPA groups, mm^2^(*N*)	Serum 25(OH)D^¶^, nmol L^−1^	Total calcium, mmol L^−1^
Men, *n* = 1994		
No plaques (949)	54.0 ± 16.4	2.37 ± 0.10
<11.37 (348)	55.7 ± 17.0	2.37 ± 0.10
11.37–23.89 (347)	55.6 ± 16.6	2.36 ± 0.10
>23.89 (348)	54.9 ± 16.9	2.37 ± 0.11
*P* for trend	NS	NS
*β* unadjusted (95% CI)^*₤*^	0.02 (−0.03, 0.07)	−0.03 (−0.08, 0.02)
*β* adjusted^†^ (95% CI)^*₤*^	0.02 (−0.03, 0.06)	−0.03 (−0.08, 0.01)

Women, *n* = 2200		
No plaques (1238)	50.3 ± 16.3	2.37 ± 0.11
<9.42 (319)	49.5 ± 18.3	2.38 ± 0.12
9.42–18.07 (321)	50.2 ± 17.7	2.39 ± 0.12
>18.07 (322)	48.6 ± 16.0	2.40 ± 0.11
*P* for trend	NS	<0.001
*β* unadjusted (95% CI)^*₤*^	−0.03 (−0.07, 0.02)	0.08 (0.04, 0.13)*
*β* adjusted^†^ (95% CI)^*₤*^	0.04 (−0.01, 0.08)	0.01 (−0.04, 0.05)

^¶^Adjusted for season of blood testing.

*P < 0.05.

^*₤*^Square root transformed TPA as the dependent variable in the linear regression model.

^†^Adjusted for age, BMI, systolic blood pressure, and total cholesterol.

**Table 4:** Odds ratios (OR) and their respective 95% confidential intervals for the association between serum 25(OH)D and levels of mean IMT and TPA in men and women, using multinomial logistic regression. The 4th Tromsø Study, 1994-1995.

Multivariate models	Men, *n* = 1994							
	Mean IMT quartiles, mm				TPA groups, mm^2^			
	<0.76	0.76–0.87	0.88–1.00	>1.00	No plaques	<11.37	11.37–23.89	>23.89
Model 1	1.00	1.11 (0.98, 1.26)	1.03 (0.90, 1.17)	1.14* (1.00, 1.30)	1.00	1.09 (0.96, 1.23)	1.09 (0.97, 1.24)	1.03 (0.91, 1.17)
Model 2	1.00	1.14 (0.99, 1.30)	1.12 (0.96, 1.30)	1.30** (1.12, 1.51)	1.00	1.06 (0.93, 1.20)	1.10 (0.96, 1.25)	1.08 (0.94, 1.23)

Multivariate models	Women, *n* = 2200							
	Mean IMT quartiles, mm				TPA groups, mm^2^			
	<0.72	0.72–0.81	0.82–0.93	>0.93	No plaques	<9.42	9.42–18.07	>18.07

Model 1	1.00	0.90 (0.80, 1.01)	0.89 (0.80, 1.01)	0.77 (0.68, 0.86)**	1.00	0.93 (0.82, 1.06)	0.98 (0.87, 1.11)	0.89 (0.78, 1.01)
Model 2	1.00	1.02 (0.90, 1.16)	1.12 (0.98, 1.28)	1.10 (0.96, 1.28)	1.00	1.00 (0.88, 1.14)	1.12 (0.98, 1.27)	1.15* (1.01, 1.33)

Model 1. Serum 25 (OH) D adjusted for season when vitamin D samples were obtained.

Model 2. Serum 25 (OH) D adjusted for season of sampling, age, BMI, SBP, and total cholesterol.

*P < 0.05.

**P < 0.001.

**Table 5:** Odds ratios (OR) and their respective 95% confidential intervals for the association between total calcium and levels of mean IMT and TPA in men and women, using multinomial logistic regression. The 4th Tromsø Study, 1994-1995.

Multivariate models	Men, *n* = 1994							
	Mean IMT quartiles, mm				TPA groups, mm^2^			
	<0.76	0.76–0.87	0.88–1.00	>1.00	No plaques	<11.37	11.37–23.89	>23.89
Model 1	1.00	0.92 (0.81, 1.03)	0.95 (0.84, 1.07)	0.91 (0.81, 1.03)	1.00	0.92 (0.82, 1.04)	0.88* (0.78, 1.00)	0.96 (0.85, 1.08)
Model 2	1.00	1.01 (0.88, 1.16)	1.04 (0.90, 1.19)	0.95 (0.82, 1.10)	1.00	0.93 (0.82, 1.05)	0.87 (0.77, 0.99)	0.92 (0.81, 1.05)

Multivariate models	Women, *n* = 2200							
	Mean IMT quartiles, mm				TPA groups, mm^2^			
	<0.72	0.72–0.81	0.82–0.93	>0.93	No plaques	<9.42	9.42–18.07	>18.07

Model 1	1.00	1.17** (1.04, 1.31)	1.27*** (1.13, 1.43)	1.38*** (1.23, 1.55)	1.00	1.02 (0.90, 1.14)	1.12* (1.00, 1.27)	1.24*** (1.10, 1.40)
Model 2	1.00	1.03 (0.90, 1.16)	1.08 (0.94, 1.23)	1.08 (0.94, 1.24)	1.00	0.92 (0.81, 1.05)	0.99 (0.87, 1.12)	1.03 (0.90, 1.17)

Model 1. Total calcium, unadjusted.

Model 2. Total calcium adjusted for age, BMI, SBP, and total cholesterol.

*P < 0.05.

**P < 0.01.

***P < 0.001.

**Table 6:** Adjusted odds ratio (OR) and their respective 95% confidential intervals for IMT >1.00 mm in men and IMT >0.93 mm in women by subgroup of serum 25-hydroxyvitamin D concentration in the whole population (including smokers)* as well as in nonsmokers^¶^. The 4th Tromsø Study, 1994-1995.

Serum 25(OH)D, nmol/L	Whole population				Nonsmokers			
	*n*	Men	*n*	Women	*n*	Men	*n*	Women
<30.0	94	0.63 (0.36, 1.12)	226	1.01 (0.65, 1.55)	78	0.64 (0.32, 1.31)	204	0.59 (0.33, 1.05)
30.0–49.9	853	0.67 (0.51, 0.89)	1101	1.10 (0.81, 1.52)	742	0.72 (0.48, 1.08)	975	0.71 (0.44, 1.16)
50.0–74.9	1466	0.81 (0.64, 1.03)	1353	1.07 (0.80, 1.43)	963	0.92 (0.62, 1.36)	878	0.71 (0.43, 1.16)
75.0–100.0	484	1.0 (Reference)	456	1.0 (Reference)	185	1.0 (Reference)	126	1.0 (Reference)
>100.0	99	0.90 (0.54, 1.49)	98	0.74 (0.40, 1.38)	26	1.77 (0.62, 4.81)	17	0.22 (0.03, 1.85)

*Adjusted for age, BMI, SBP, season for vitamin D sampling, total cholesterol, and smoking status.

^¶^Adjusted age, BMI, SBP, season for vitamin D sampling, and total cholesterol.
